# Clinical effectiveness of the PolyScope™ endoscope system combined with holmium laser lithotripsy in the treatment of upper urinary calculi with a diameter of less than 2 cm

**DOI:** 10.3892/etm.2013.1184

**Published:** 2013-06-25

**Authors:** SI-PING GU, YUN-TENG HUANG, ZHI-YUAN YOU, XIAOMING ZHOU, YI-JIN LU, CAO-HUI HE, JUAN QI

**Affiliations:** 1 Micro-Invasive Surgery Center, Huaqiao Hospital, Shishi, Fujian 362700;; 2 Department of Urology, Xinhua Hospital Affiliated to Shanghai Jiao Tong University, Shanghai 200092;; 3 Department of Minimally Invasive Surgery, Wanxiang Minimally Invasive Hospital, Quanzhou, Fujian 362000;; 4 Department of Urology, Micro-Invasive Surgery Center, The First Hospital Affiliated to Guangzhou Medical University, Guangzhou, Guangdong 510120, P.R. China

**Keywords:** modular, flexible ureteroscope, Holmium laser, lithotripsy, urinary calculi

## Abstract

The aim of this study was to evaluate the clinical value of the PolyScope™ endoscope system in the treatment of upper urinary calculi with a diameter of <2 cm. A total of 86 patients hospitalized with upper urinary tract calculi were included. The patients were placed under general or spinal anesthesia and in a lithotomy position. Following the dilation of the ureter, a guide wire was inserted under the direct vision of an F8/9.8 rigid ureteroscope, and an F12/14 flexible ureteral access sheath was positioned along the guide wire. Holmium laser lithotripsy was subsequently performed, using an F8.0 ‘PolyScope’ modular flexible ureteroscope. Plain film of the kidney-ureter-bladder (KUB) was performed 1 day subsequent to the surgery, in order to determine the result of the lithotripsy and the position of the double-J stent which was inserted after after holmium laser lithotripsy. In addition, in certain patients, KUB radiography was performed 2–4 weeks subsequent to the surgery, and extracorporeal shockwave lithotripsy (ESWL) was performed if the diameter of the residual stones was >6 mm. Lithotripsy was successful in 77 patients and the duration of the surgery ranged between 25 and 80 min (mean duration, 42 min). Little bleeding was observed. Three patients presented with a slight fever following the surgery; however, no ureteral perforation, high fever or septicemia was observed among the patients following anti-inflammatory treatment. The stone-free rate (SFR) of the single-pass lithotripsy was 89.5% (77/86) and the SFR with ESWL was 96.5% (83/86). The study demonstrated that the F8 modular flexible ureteroscope was safe, convenient and effective for the lithotripsy of upper-tract calculi.

## Introduction

The flexible ureteroscope has been widely used by numerous urologists in the management of upper urinary tract calculi (including renal and ureteral stones), due to its desirable therapeutic effect. The ureteroscopy process uses the natural channels of the human body, making it minimally invasive, and provides an essentially unhindered view throughout. However, the traditional one-piece flexible ureteroscope is expensive and vulnerable during surgery. In addition, it has high maintenance costs and a long repair cycle. Therefore, the German company, Polydiagnost GmbH (Pfaffenhofen, Germany), has developed a new, high-end, minimally invasive modular flexible ureteroscope (PolyScope™). This removable modular flexible ureteroscope has core components, such as the optical and imaging systems, which have been designed as independent parts and are convenient to assemble and disassemble. The optical system of the PolyScope utilizes single optical fiber technology, and has a soft and tough synthetic metal wrapped around the surface for protection. During its use, it is placed into the fiber channel of the lens barrel. The PolyScope produces stable and clear images; thus, it has been suggested to be an effective solution for overcoming the shortcomings of the conventional flexible ureteroscope (1). In the present study, 86 patients with upper urinary tract calculi with a diameter of <2 cm were treated with the modular flexible ureteroscope in the Xinghua Hospital Affiliated to Shanghai Jiao Tong University (Shanghai, China). The results were satisfactory and are discussed in the following sections.

## Materials and methods

### Patients

A total of 86 patients (53 males and 33 females, aged from 20 to 86 years) with upper urinary tract calculi who were admitted to the Xinghua Hospital Affiliated to Shanghai Jiao Tong University from November 2010 to May 2012 were recruited for this study. Thirty-nine patients presented with upper ureteral calculi, eight with renal pelvic calculi, three with upper calyceal calculi, four with middle calyceal calculi, 21 with lower calyceal calculi, eight with ureteral and lower calyceal calculi, one with ureteral and middle calyceal calculi, one with renal pelvic and lower calyceal calculi, and one with middle and lower calyceal calculi. In addition, 45 patients exhibited left-sided calculi, whereas 41 patients exhibited right-sided calculi. The mean diameter of the calculi was 1.23±0.27 cm. Nineteen patients (11 patients with ureteral stenosis, six with urinary tract infection and two with renal insufficiency) had previously had an indwelling double-J stent, placed with ureteroscopic guidance, for a duration of 2–8 weeks. The major clinical manifestations of the patients were ipsilateral lower back pain, abdominal pain, urinary tract infection and gross or microscopic recurrent hematuria. The majority of the patients had been hospitalized and undergone surgery following conservative treatment, such as treatment against infection; however, the extra water and medical expulsive therapies failed to cure the symptoms. The case inclusion criteria for the present study were as follows: The distance from the pelvis to the upper ureteral calculi was <5 cm; the diameter of the stones was <2.0 cm in the pelvis and the upper and middle calyces; the diameter of the stones was <1.5 cm in the lower calyx; and the patients were without combined surgical contraindications. The patients and their relatives were briefed with regard to the details of the surgical intervention for the upper urinary tract calculi, the choice of surgical approach, the advantages and disadvantages of flexible ureteroscopy combined with holmium laser lithotripsy and the surgical risks. This study was conducted in accordance with the Declaration of Helsinki and with approval from the Ethics Committee of Xinhua Hospital Affiliated to Shanghai Jiao Tong University. Written informed consent was obtained from all participants.

### Methods

The patients were placed under general or spinal anesthesia and in a lithotomy position. Having located the ipsilateral ureter, a zebra guide wire was inserted and a Wolf F8/9.8 ureteroscope (Richard Wolf GmbH, Knittlingen, Germany) was placed into the ureter so as to reach the level of the pelvis and determine the approximate distribution of the intrarenal calyceal. Following the removal of the ureteroscope, a Cook F12/14 Ureteral Access Sheath (UAS; Cook Medical, Inc., Bloomington, IN, USA) was positioned along the guide wire. The UAS was moved as close to the level of the ureteropelvic junction as possible. The Polydiagnost F8 modular flexible ureteroscope (Polydiagnost GmbH) was placed into the pelvis along the UAS. A 220 mm holmium laser lithotripsy with a power setting of 1.0 J at 10–20 Hz was used when stones were encountered. The ‘nibble’ approach was used to break and crumble the stones into fine granules measuring 2–3 mm, in order to avoid prolonging the surgery unnecessarily while performing repeated searches for stones that had been broken into large pieces. Stones measuring ≤4 mm were removed with a stone basket and some lower calyceal stones were trans-located with the stone basket from the lower to the upper or middle calyx, in order to improve the efficiency of the stone breaking. An F6 double-J stent was typically indwelling for 4 weeks following the surgery and a Foley’s urethral catheter was indwelling for 1–7 days. Plain film of kidney-ureter-bladder (KUB) was performed 1 day subsequent to the surgery, in order to determine the result of the lithotripsy and the position of the double-J stent. However, in certain patients, KUB radiography was performed 2–4 weeks following the surgery. Extracorporeal shockwave lithotripsy (ESWL) was performed if the diameter of any residual stones was >6 mm.

## Results

**Figure 1.**
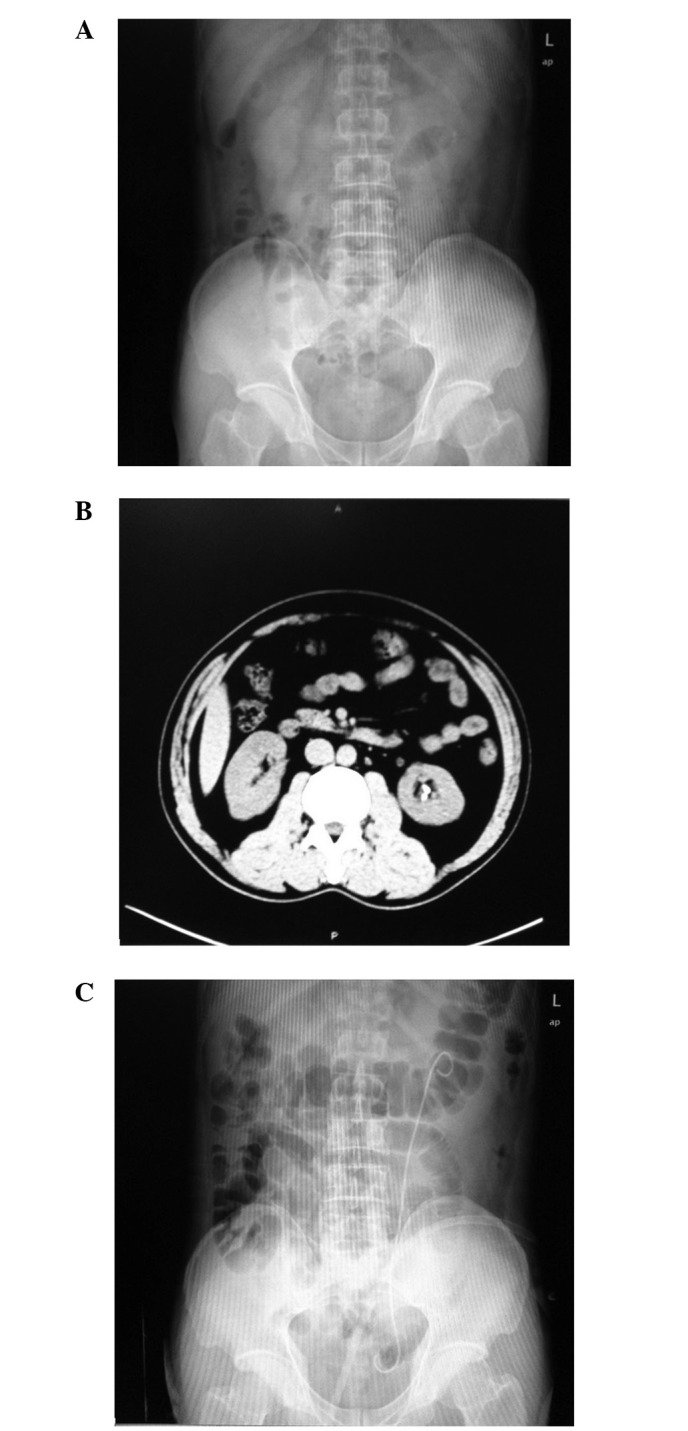
(A) Preoperative kidney, ureter and bladder (KUB) radiograph of Case 1, showing two stones in the lower calyx of the left kidney (diameter 6–7 mm). (B) Preoperative computed tomography (CT) scan showing two stones in the lower calyx of the left kidney (diameter 6–7 mm). (C) KUB radiograph of Case 1 at day 1 postoperatively, indicating that the two stones were completely removed.

**Figure 2.**
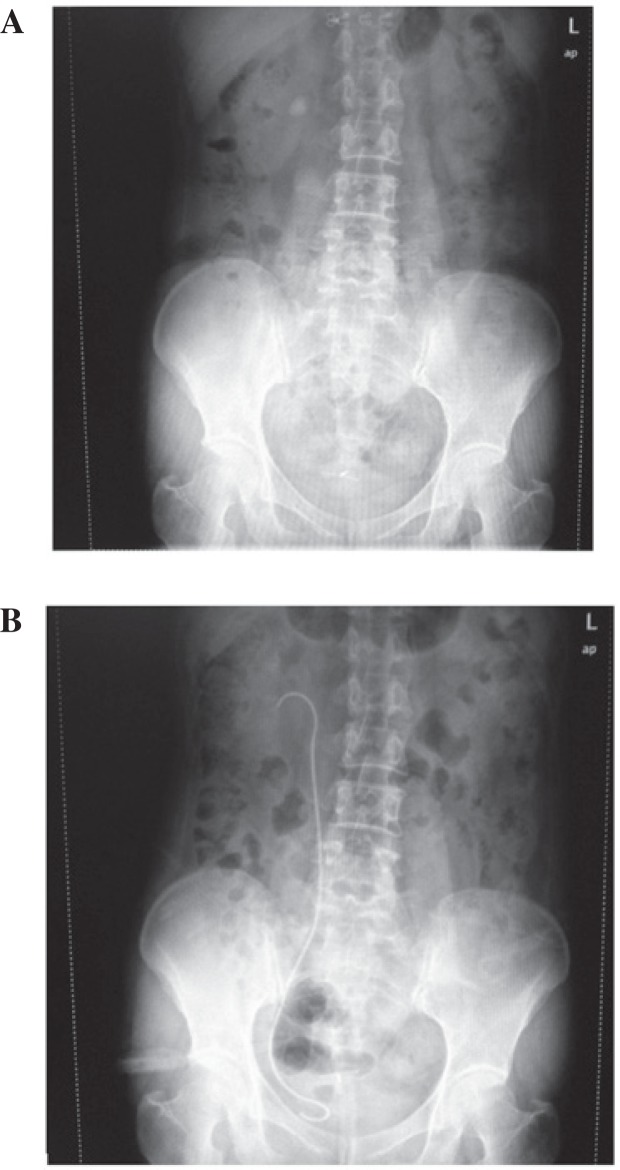
(A) Preoperative kidney, ureter and bladder (KUB) radiograph of Case 2, showing a stone at the right pelvis (diameter 1.8 cm). (B) KUB radiograph of Case 2 at day 1 postoperatively, indicating that the stone at the right pelvis was completely removed.

**Figure 3.**
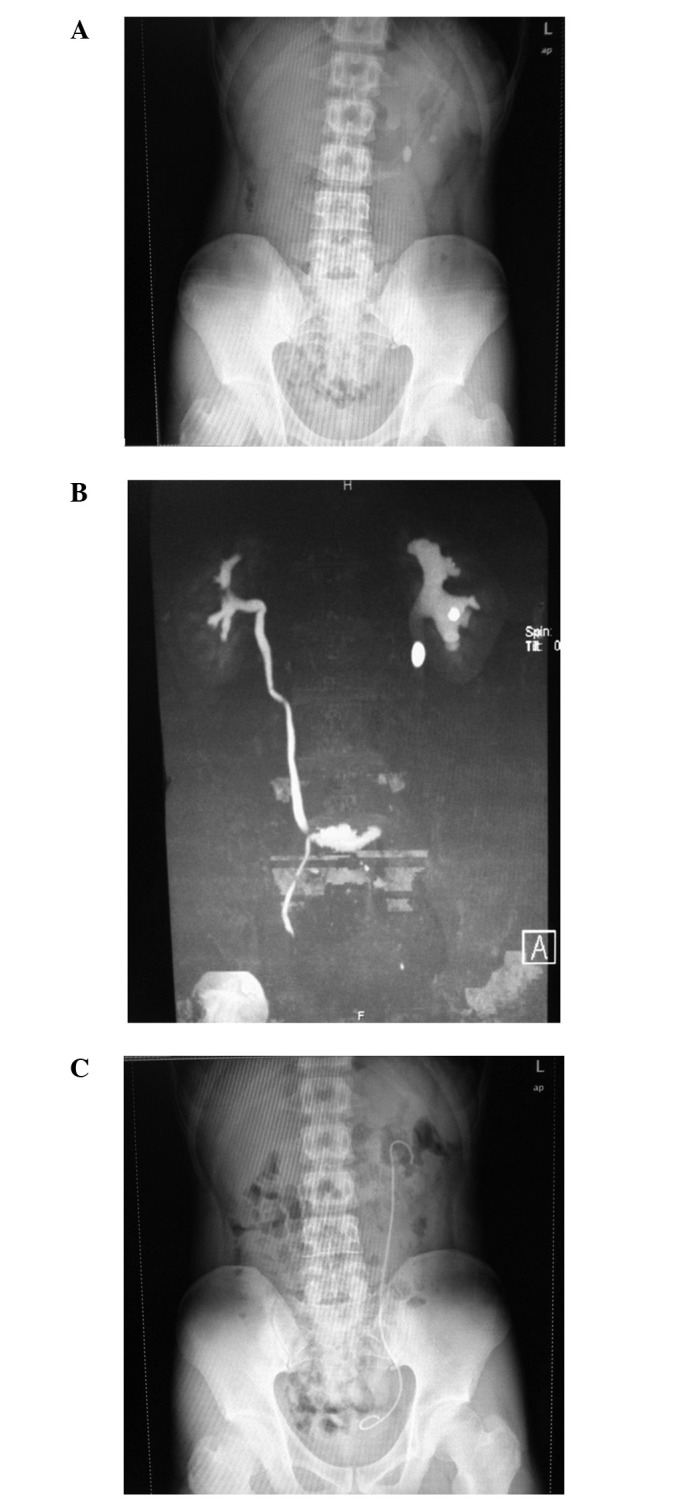
(A) Preoperative kidney, ureter and bladder (KUB) radiograph of Case 3, showing a stone in the upper left ureter (diameter 1.7 cm) and a stone in the left middle calyx (diameter 0.8 cm). (B) Preoperative computed tomography urography (CTU) scan of Case 3, showing a stone in the upper left ureter (diameter 1.7 cm) and a stone in the left middle calyx (diameter 0.8 cm). (C) KUB radiograph of Case 3 at day 1 postoperatively, indicating that the two stones were completely removed.

Lithotripsy was performed successfully in 77 patients. The duration of the surgery ranged from 25 to 80 min (mean duration, 42 min). Little bleeding was recorded. Three patients presented with a slight fever following the surgery; however, no ureteral perforation, high fever or septicemia was observed among the patients following anti-inflammatory treatment. The stone-free rate (SFR) of the single-pass lithotripsy was 89.5% (77/86). Among the remaining nine patients, three patients with upper ureteral calculi and one with calyceal calculi exhibited postoperative residual stones and underwent ESWL; in addition, one patient underwent percutaneous nephrolithotomy (PCNL) due to a narrow and distorted ureteral structure. Among the four cases with lower calyceal calculi, three underwent ESWL, as the angle of the funnel pelvis was too narrow (two cases were successful and one case had residual stones due to the hardness of the calculi). One patient with lower calyceal calculi did not undergo further procedures, since the patient’s kidney exhibited severe hydrocephalus and location of the stones was not possible. Therefore, the SFR with ESWL was 96.5% (83/86). The postoperative follow-up period was 1–3 months. No residual fragments measuring >3 mm remained following the reviews with B-mode ultrasound or radiography. The longest stone expulsion time was 7 weeks (Figs. 1–3).

## Discussion

Calculus of the urinary tract is a common disease requiring surgical intervention. According to global statistics, between 5 and 15% of the population suffer from urinary stone disease (2). The use of lithotripsy with a flexible ureteroscope for the treatment of renal or ureteral stones demonstrates numerous advantages, including minimal invasiveness, safety, good efficacy, little pain, rapid recovery and direct visibility; thus, an increasing number of urologists are inclined to use flexible ureteroscopy to manage renal or ureteral stones. According to the present relevant guidelines on urolithiasis treatment in Europe and the United States and from the China Urology Association, the main international indications for flexible ureteroscopy in the treatment of renal stones are: X-rays are negative for stones, leading to difficulties with ESWL positioning (<2 cm); the presence of residual lower calyceal stones following ESWL surgery; incarcerated lower calyceal stones, resulting in a poor efficacy with ESWL; the presence of hard stones (such as calcium oxalate monohydrate, or cystine calculi) which are unsuitable for ESWL treatment; extremely obese patients or individuals with a serious spinal deformity, in whom there may be difficulties establishing PCNL; and the presence of intradiverticular calyceal stones accompanied by calyceal neck stenosis. In addition, other special cases also exist, such as combined hemorrhagic diathesis, horseshoe kidney, pelvic ectopic kidney, complex renal anatomy, solitary renal calculi, lower calyceal calculi <2 cm and children with renal calculi <1.5 cm. Even renal calculi measuring between 2 and 4 cm have been recommended for treatment using flexible ureteroscopic lithotripsy (3–11). Therefore, the effects of flexible ureteroscopic lithotripsy have been demonstrated to be approximately equal to those of PCNL, but higher than those of ESWL, while the surgical risks of flexible ureteroscopic lithotripsy have been demonstrated to be lower than those of PCNL. Furthermore, the convalescence has been shown to be more rapid for flexible ureteroscopic lithotripsy than PCNL and the risk of long-term complications has been shown to be lower than that for ESWL (12,13). Compared with ESWL and PCNL, flexible ureteroscopy has the advantages of being minimally invasive, exhibiting a desirable efficacy and resulting in fewer complications, thus making it more acceptable for doctors and patients. Therefore, with the increase in risk awareness shown by doctors and patients, economic development and the widespread focus on health, the use of a flexible ureteroscope combined with a holmium laser in the treatment of urinary stones has gradually become one of the main approaches for lithotripsy (4,7,8). However, the loss and maintenance of flexible ureteroscopes has been a major challenge in clinical practice. At present, the flexible ureteroscope commonly used in clinical practice is expensive and has a high maintenance cost and long maintenance cycle, which limit the application of flexible ureteroscopy. It has been suggested that flexible cystoscopes undergo major damage due to the passing of certain tools, such as optical fibers and stone extraction forceps, through the working channel (14). Afane *et al* (15) assessed a selection of 9Fr flexible utero-scopes, including the Storz model 11274AA, the double-dagger Circon-ACMI model AUR-7 and the Wolf 7325.172 model, and observed that it was necessary for the uteroscopes to be maintained following 6–15 surgeries and 3–12.8 h of surgical time.

The removable modular flexible ureteroscope is similar in shape to the traditional flexible ureteroscope; however, the camera optical fiber, imaging systems and other core components are provided as independent components. Consumable components, such as the endoscope, may be removed and replaced easily. The most valuable component is the optical system, which is adapted from the technology of a single-mode optical fiber. A metal alloy surrounds the fiber surface for protection. When the optical system is utilized, it is placed into the endoscopic fiber channel. Unlike the traditional quartz collection fiber structure, it has 10,000 pixels, and is able to detect targets with a diameter of ∼0.125 mm within a distance of 2–4 mm, and is able to transfer clear and stable images without spots, grids or honeycomb artifacts. The working channel of the removable modular flexible ureteroscope is 3.6 F, which is similar to that of a traditional flexible ureteroscope. In an empty working channel, the lens barrel has been demonstrated to bend at an angle of 265°; the bending angle is reduced by 10 and 2%, respectively, following the insertion of a 3.0F stone basket or a 200 *μ*m laser fiber (1). At a perfusion pressure of 100 mmHg, the perfusion flow rate has been reported to decrease by 50 and 70% (to 28.5 ml/min and 18.3 ml/min) following the insertion of 200 *μ*m and 365 *μ*m fibers, respectively. These conditions meet the basic visual demand of a flexible ureteroscope with a 10 ml/min flow rate (1,16).

In the present study, the modular flexible ureteroscope was used to treat 86 cases of kidney and ureter lithiasis, involving stones located in the renal pelvis, the upper, middle and lower calyces and the upper ureter. The overall effect was satisfactory with a single-pass SFR of 89.5% (77/86), a second-pass SFR of 96.5% (83/86), an upper ureteral calculi SFR of 89.7% (35/39) and a calyceal calculi SFR of 87.1% (27/31). With regard to the use of a modular flexible ureteroscope combined with a holmium laser for the treatment of upper urinary calculi in these 86 cases, there were several points of note. It was realized that, prior to the insertion of the modular flexible ureteroscope, it was necessary for the UAS of the flexible ureteroscope to be indwelling and for the end of the UAS to be placed near the level of the ureteropelvic junction (on encountering ureteral stenosis, it was not possible to perform a single-pass placement of the UAS in the ureter; in such cases, an F6 double-J stent was indwelling for a second lithotrity). The indwelling UAS helped to reduce the renal pelvic pressure during surgery. Rehman *et al* (17) observed in their experiments that, using a UAS, the renal pelvic pressure (RPP) was <20 cm H_2_O when the liquid perfusion pressure was 200 cm H_2_O. Maintaining the low pressure state of RPP may help to reduce the intraoperative absorption of lavage fluid and the incidence of postoperative fever and bacteremia. The indwelling UAS provided a convenient access point and served as protection for the lens barrel, which was beneficial for the extraction of stone fragments. Sanguedolce *et al* (18) compared cases with an indwelling UAS (n=66) and those with a non-indwelling UAS (n=74) and observed that there were no significant differences in the duration of the surgery, SFR or the incidence of postoperative complications. In addition, in a long-term follow-up (average follow-up, 25 months), the UAS indwelling group had no long-term complications of ureteral stricture.

An additional factor demonstrated in the present study was that, following the placement of the flexible ureteroscope into the renal pelvis, it was necessary to initially distinguish the renal ureteropelvic junction and set it as an index point. It was then possible to identify the positions of the renal upper, middle and lower calyces gradually to locate the stones. Following the location of the stones, it was necessary for the end of the flexible ureteroscope to be maintained in a stretched state during the insertion of the 220 *μ*m holmium laser optical fiber. Subsequent to the appearance of the fiber in the field of vision, the optical fiber was retracted into the body by 1–2 mm. Once the target stones were re-identified, the laser optical fiber was extended out of the mirror body by 3–4 mm to conduct the lithotripsy. The laser displacement device of the flexible ureteroscope kit was selected when necessary to fix the optical fiber and prevent the fiber from rebound damage during the process. This method aided the protection of the work channel of the lens barrel and the lens, and prevented the laser optical fiber from causing mechanical damage to the kidney.

In the process of lithotripsy, the setting power of the holmium laser lithotripsy was 0.8–1.0 J/5–10 Hz. It was possible to adjust the frequency to 15–20 Hz, depending on the circumstances, in order to enhance the effect. The process started from the surrounding area and adopted the ‘nibble’ approach. A ‘middle drilling’ method was initially performed to break the larger pelvic or renal calyceal stones in a sequential fashion. It was observed that it was necessary to crush the stones into fragments of <4 mm for easy excretion. The modular flexible ureteroscope exhibited a unidirectional turning design, which caused it to be less convenient than the traditional flexible ureteroscope. In the search for calculi and fragments, rotation and cooperation of the lens barrel and arms was required. It was possible to lose the sense of direction in the intracavity as a result of the process of turning the lens barrel; in such situations, it was possible to extract the mirror from the body to reorient it. However, the steering amplitude of the bottom of the lens was proportional to the degree of the crimping of the operating handle, since the handle portion of the lens barrel was made of plastic. Therefore, the handle broke easily when pinched excessively, thereby increasing the losses in the flexible ureteroscope kit (19).

Modular flexible ureteroscopy has the advantages of simple operation, clear visibility and a reliable efficacy. When the optical fiber is inserted, a bending angle of >180° was achievable, which was sufficient for the clinical needs. Compared with the conventional optical or electronic flexible ureteroscope, as single-use designs, the biggest weakness of this type of flexible ureteroscope is the unidirectional turning design, which results in it only being possible to turn the ureteroscope in one direction. Rotation and cooperation of the mirror body and the arms was necessary during surgery. The sense of direction in the intracavity may be lost when turning the mirror body, and this prolongs the duration of the surgery and potentially increases the risk of intraoperative and postoperative complications. Certain limitations in the mirror dexterity, functionality, directional grasp and operational handling remain. Moreover, the vulnerability of the flexible ureteroscope may not be fundamentally reduced. However, there are numerous advantages with the clinical application of the modular flexible ureteroscope. The core components (such as the optical imaging fiber) are split-type, well-protected and supported out of the box, while parts that become worn (such as the mirror body) may be replaced individually, which greatly reduces the costs, since there is no requirement to pay for equipment repair and maintenance. The outer casing of the flexible ureteroscope is replaceable at a low price and the components are disposable. This avoids the risk of cross-infection and enables the flexible ureteroscope to be used in successive procedures (20,21). Modular flexible ureteroscopy is applicable in cases with large upper urinary tract calculi, for surgeries with a long duration and for cases with large losses of lenses. This is likely to facilitate the popularization and application of flexible ureteroscope technology.

However, due to the limited number of cases involved in the present study, the use of the PolyScope endoscope system requires further study, for example, in studies concerning the use of flexible ureteroscopes for the treatment of renal calyceal calculi or renal calculi with a diameter >2 cm, and regarding the combined use of a flexible ureteroscope with a percutaneous nephroscope for the treatment of certain complex renal calculi.
